# Bilateral Reverse Total Shoulder Arthroplasty for Bilateral Proximal Humeral Fractures Complicated by Periprosthetic Fractures

**DOI:** 10.7759/cureus.90429

**Published:** 2025-08-18

**Authors:** Jin Nagasawa, Yoshihiro Hirakawa, So Taniguchi, Tomoya Manaka

**Affiliations:** 1 Orthopaedic Surgery, Ishikiriseiki Hospital, Higashi-Osaka, JPN; 2 Orthopaedic Surgery, Osaka Metropolitan University Graduate School of Medicine, Osaka, JPN

**Keywords:** bilateral shoulder injury, osteoporosis, periprosthetic fracture, proximal humerus fracture, reverse shoulder arthroplasty, revision joint replacement, teriparatide administration

## Abstract

Reverse shoulder arthroplasty (RSA) is being increasingly employed for complex proximal humeral fractures (PHFs) in older patients, particularly in cases with severe osteoporosis. However, bilateral PHFs and subsequent periprosthetic fractures remain rare and challenging. We report a case of a 60-year-old woman with alcoholic liver disease who sustained bilateral PHFs after a fall. Due to her compromised condition, staged bilateral RSA was performed. The initial postoperative course was uneventful. Eight months after the first surgery, she sustained a displaced periprosthetic fracture on the right, which required revision RSA with a cemented long-stem prosthesis. Two months later, a minimally displaced fracture occurred on the left, accompanied by radiographic loosening. Conservative management with teriparatide achieved successful bone union. At final follow-up, shoulder function declined on the revised side but was preserved on the conservatively managed side. These findings suggest that conservative management may be a viable option when fracture displacement is limited and pain is tolerable. Moreover, early pharmacologic intervention and attention to implant technique may reduce the risk of periprosthetic complications. This case highlights the importance of individualized treatment planning for periprosthetic fractures following RSA and supports the potential role of teriparatide in promoting fracture healing.

## Introduction

Reverse shoulder arthroplasty (RSA) has recently been recognized as a reliable treatment option for complex proximal humeral fractures (PHFs), particularly in older patients [1,2]. In patients with poor bone quality, achieving tuberosity healing with treatments such as open reduction and internal fixation (ORIF) is often difficult. RSA reportedly results in fewer reoperations compared to ORIF [3,4] and, therefore, has become a primary treatment option for such injuries. PHFs are predominantly caused by trauma and typically occur unilaterally. However, instances of bilateral RSA performed for simultaneous bilateral fractures in patients with severely compromised bone quality or who have atypical fall patterns have been reported [5]. Furthermore, periprosthetic fractures following RSA are recognized as significant complications in older patients with poor bone quality. This highlights the need for a clear classification system and treatment algorithms [6]. In this report, we present a rare case of staged bilateral RSA performed for PHFs complicated by subsequent bilateral periprosthetic fractures and discuss its clinical course and treatment strategy.

## Case presentation

**Figure 1:**
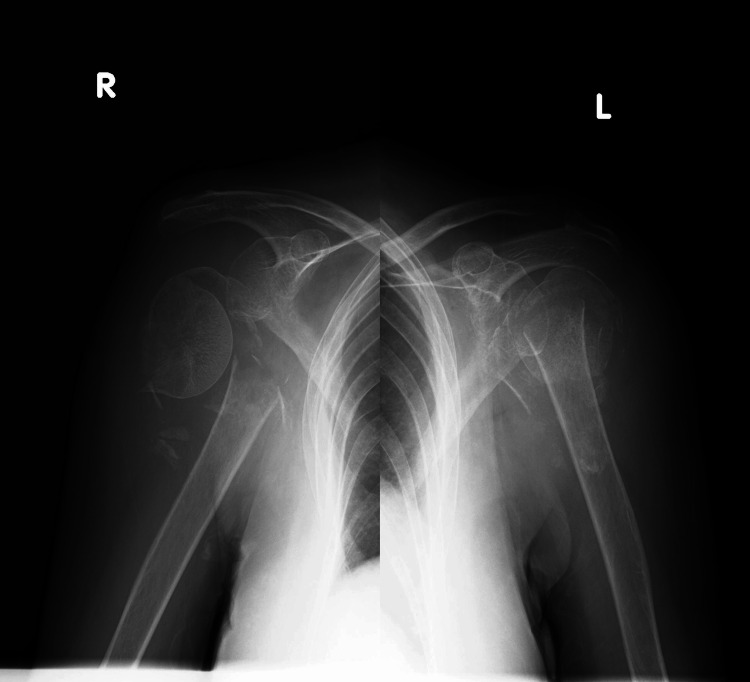
Preoperative radiograph of both shoulders Preoperative anteroposterior X-ray images of both shoulders.

A 60-year-old independent woman with a history of heavy alcohol consumption (approximately 108 g of ethanol daily) and alcoholic liver disease sustained bilateral PHFs following a fall at home. Due to mobility limitations, she remained untreated for three weeks. Upon visiting a local clinic, she was diagnosed with bilateral PHFs and referred to our institution. The initial examination revealed severe bilateral shoulder pain (worse on the right side), no neurological deficits, and preserved deltoid function. Computed tomography (CT) scans showed complex bilateral three- and four-part fractures (PHFs; right: AO/OTA 11-C3, left: AO/OTA 11-C2) (Figure 1).

Preoperative bone mineral density assessment indicated severe osteoporosis. Imaging and laboratory evaluations revealed decompensated liver cirrhosis, with jaundice and lower extremity edema. Table 1 lists key laboratory parameters often observed in patients with liver cirrhosis, including measured values, reference ranges, and abnormalities. These findings suggest hepatic dysfunction, impaired protein synthesis, or portal hypertension.

**Table 1:** Patient laboratory data A/G ratio: albumin/globulin ratio; ALP: alkaline phosphatase; ALT (GPT): alanine aminotransferase (glutamate pyruvate transaminase); AST (GOT): aspartate aminotransferase (glutamate oxaloacetate transaminase); BMD: bone mineral density; γ-GTP: gamma-glutamyl transpeptidase

Parameter	Result	Reference range
Bone mineral density	58% (femur)	≧80%
AST (GOT)	39.2	13-30 U/L
ALT (GPT)	69.1	10-42 U/L
ALP	494	104-338 U/L
γ-GTP	66	10-47 U/L
Albumin	3.4	4.0-5.0 g/dL
A/G ratio	0.86	1.2-2.0
Platelet count	10.6	130-350 × 10^3^/μL

**Figure 2:**
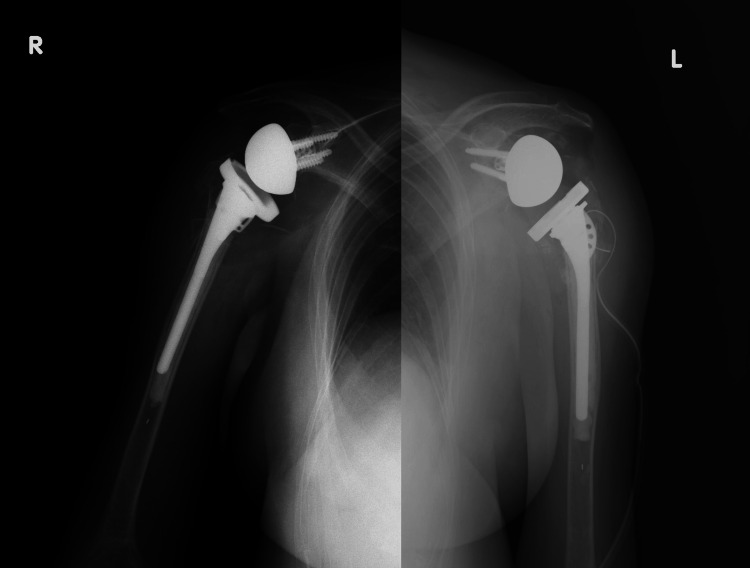
Postoperative radiographs following bilateral reverse shoulder arthroplasty Postoperative anteroposterior X-ray images of both shoulders after reverse shoulder arthroplasty.

Given the complexity of the injury and the patient’s compromised general condition, staged RSA was planned to reduce surgical risk. The procedure was performed on the right side first, followed by the left two weeks later. Both sides were treated using the Equinoxe Reverse Shoulder System (Exactech, Gainesville, FL) (Figure 2).

**Figure 3:**
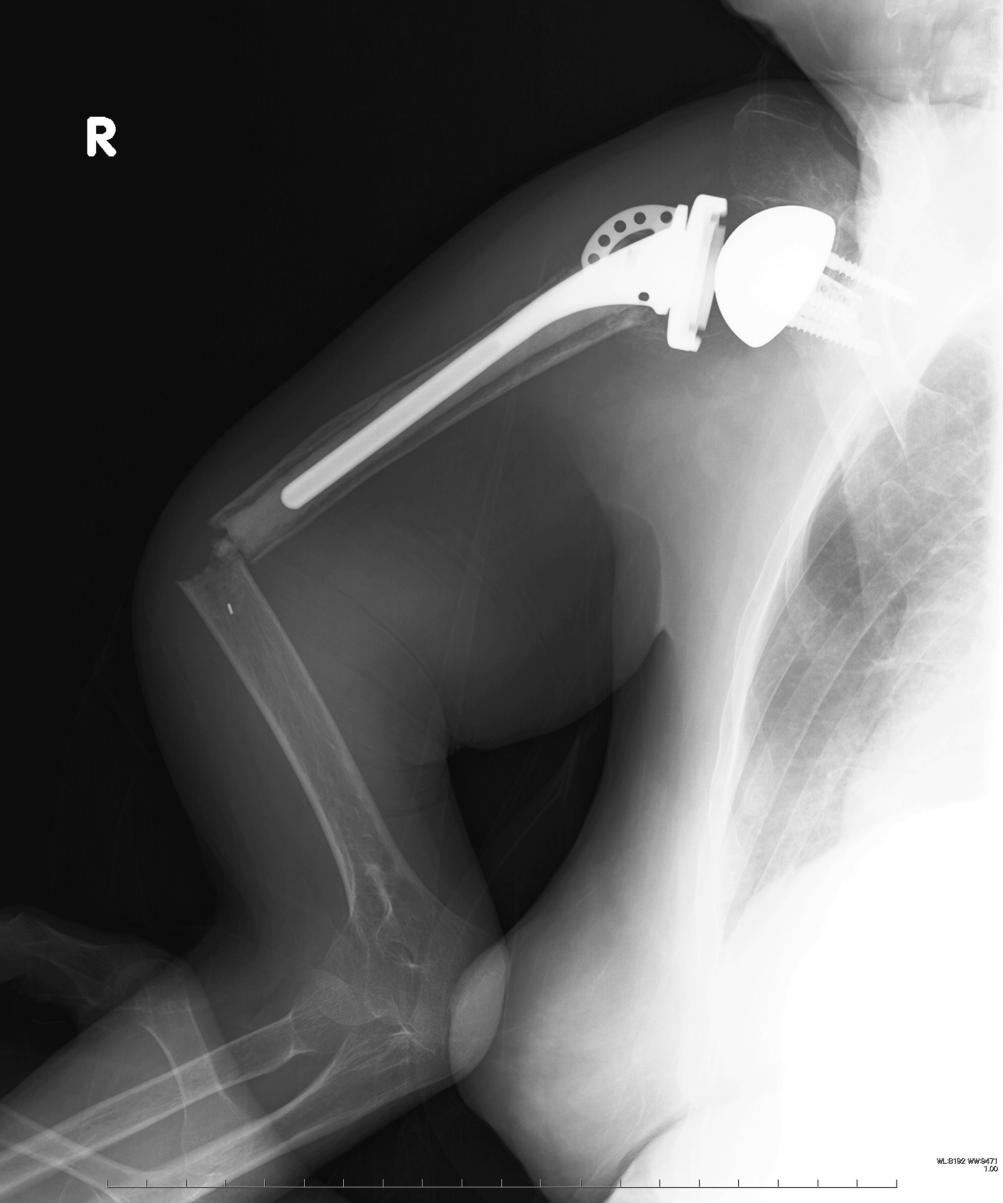
Anteroposterior plain radiograph at eight months after reverse shoulder arthroplasty A periprosthetic fracture is observed at the distal tip of the cement.

The initial postoperative recovery was reasonable, and the six-month follow-up was uneventful. However, at eight months after the primary surgery, the patient experienced another fall, resulting in a right-sided periprosthetic fracture around the humeral stem (Figure 3).

**Figure 4:**
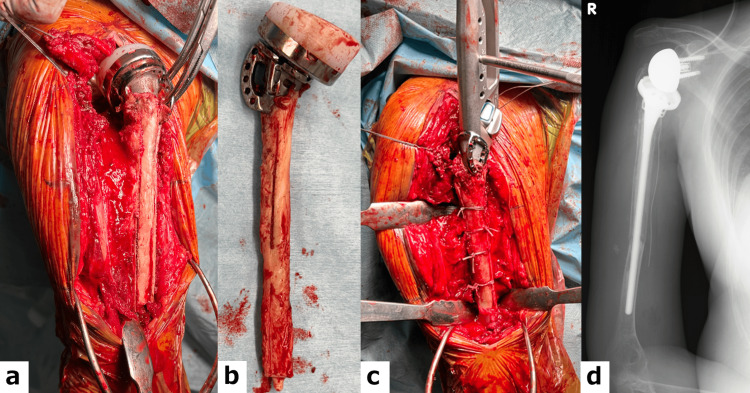
Intraoperative findings and postoperative radiograph (a) The humeral shaft was exposed using the window technique. (b) The removed STD stem showed no signs of osseous integration. (c) The cortical window was stabilized using cerclage wiring, and a long-stem prosthesis was implanted using a cement fixation technique. (d) Postoperative anteroposterior radiograph of the right shoulder showing a long-stem cemented reverse shoulder prosthesis after revision surgery.

Due to displacement and stem instability, revision RSA was performed using a cemented long stem (Figure 4).

**Figure 5:**
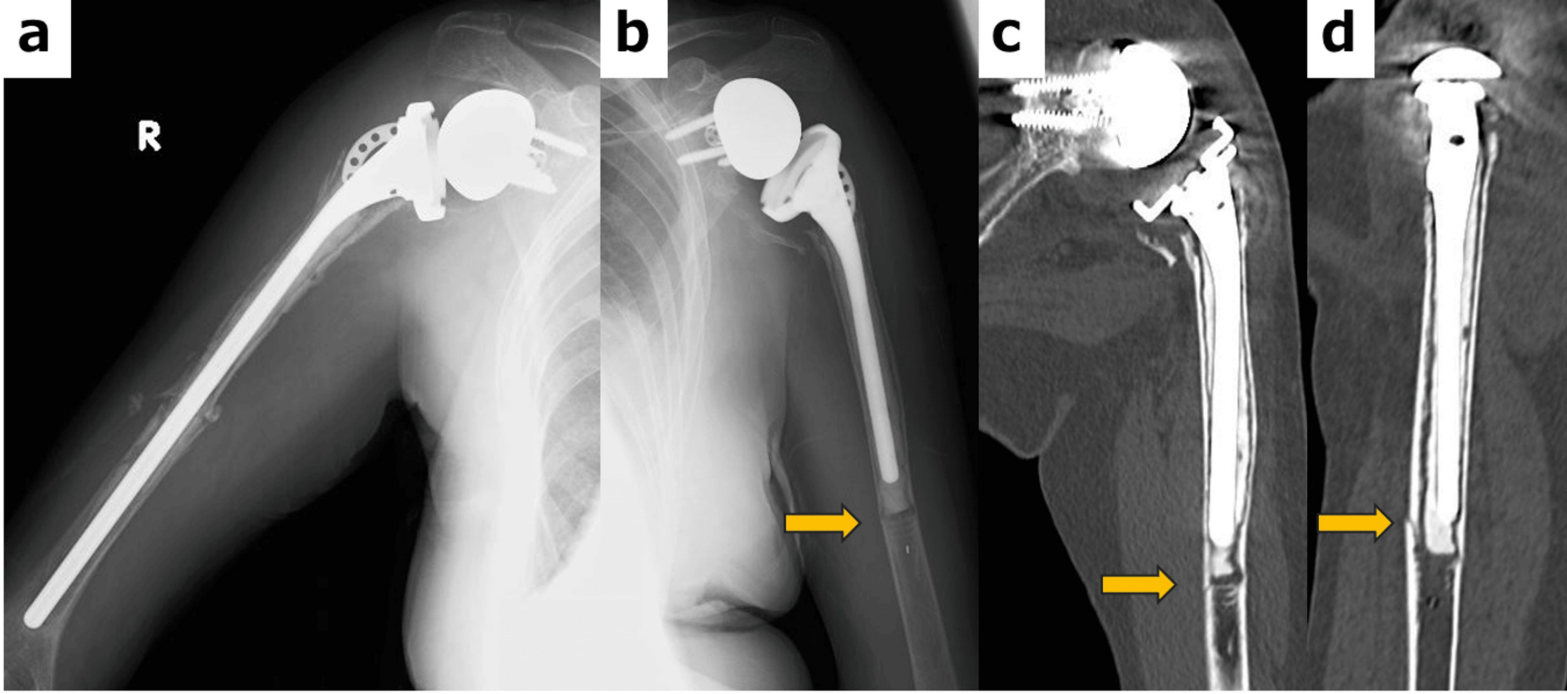
Radiographic findings at three months after right revision reverse shoulder arthroplasty and after the onset of pain in the left shoulder following reverse shoulder arthroplasty (RSA) (a) Three months after revision surgery. (b-d) A periprosthetic fracture at the distal tip of the humeral stem is visible on both X-ray and CT images at the location indicated by the arrows, accompanied by a radiolucent line around the cement mantle.

At the three-month follow-up after revision RSA on the right side (Figure 5a), the patient reported pain in the left shoulder that had started one week earlier following a new fall. Plain radiography revealed a fracture, and a CT was subsequently performed.

**Figure 6:**
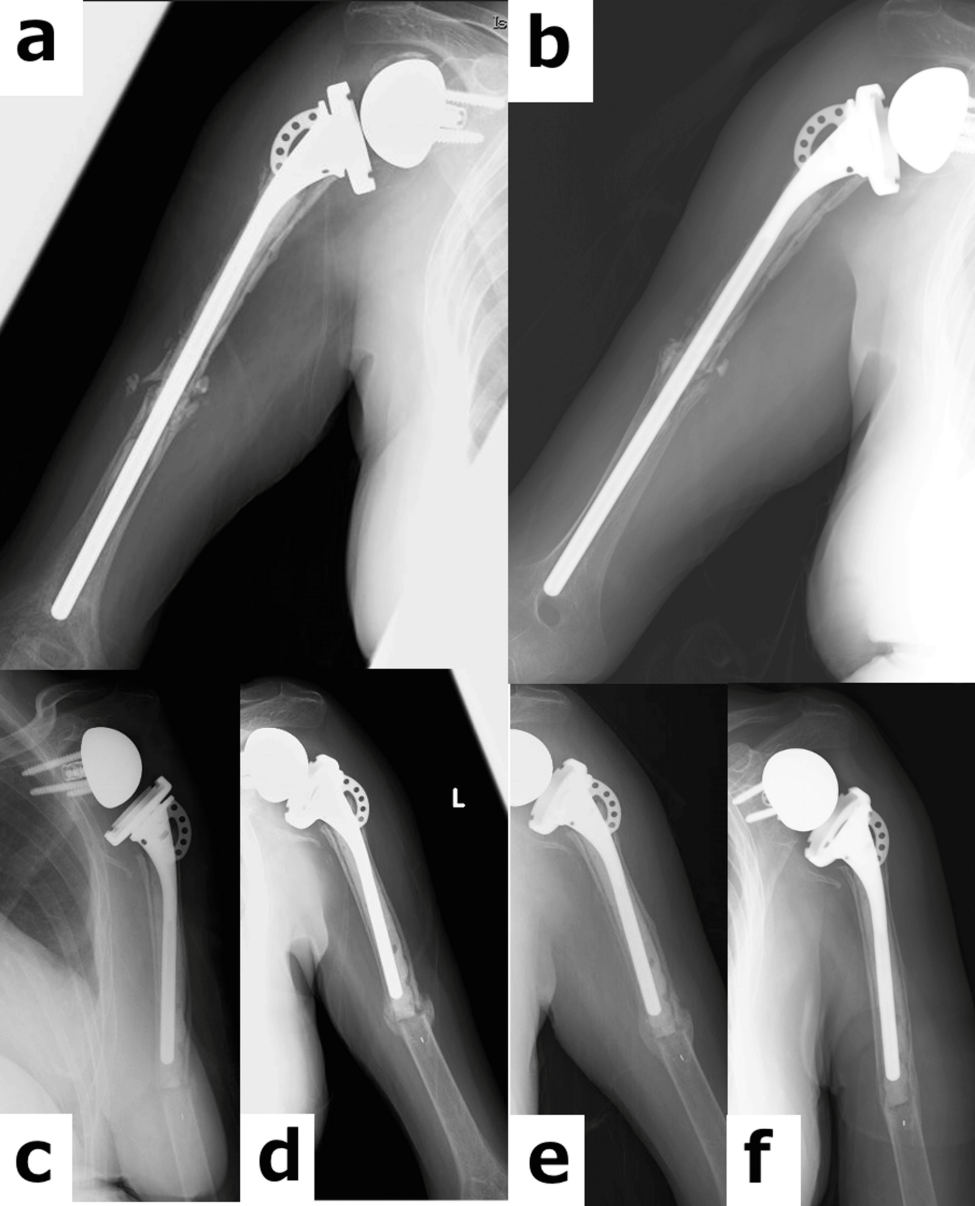
Radiographic follow-up at both shoulders (a) Radiograph at six months after revision surgery. (b) Radiograph at 15 months after revision surgery. (c) Radiograph at one month after re-injury. (d) Radiograph at three months after re-injury. (e) Radiograph at six months after re-injury. (f) Radiograph at 12 months after re-injury.

A fracture and radiolucent loosening zone around the cement mantle were also observed in the left humerus (Figures 5b-5d). After discussing treatment options with the patient, conservative management was selected. Teriparatide therapy with sling immobilization for three weeks was initiated on the same day. Although the loosening at the fracture site may have been minimal and not have affected the treatment decision, the bone union progressed favorably following administration of teriparatide. Three months after initiating conservative treatment for the left humeral fracture, robust callus formation was observed, and complete bone union was achieved at six months (Figure 6).

**Figure 7:**
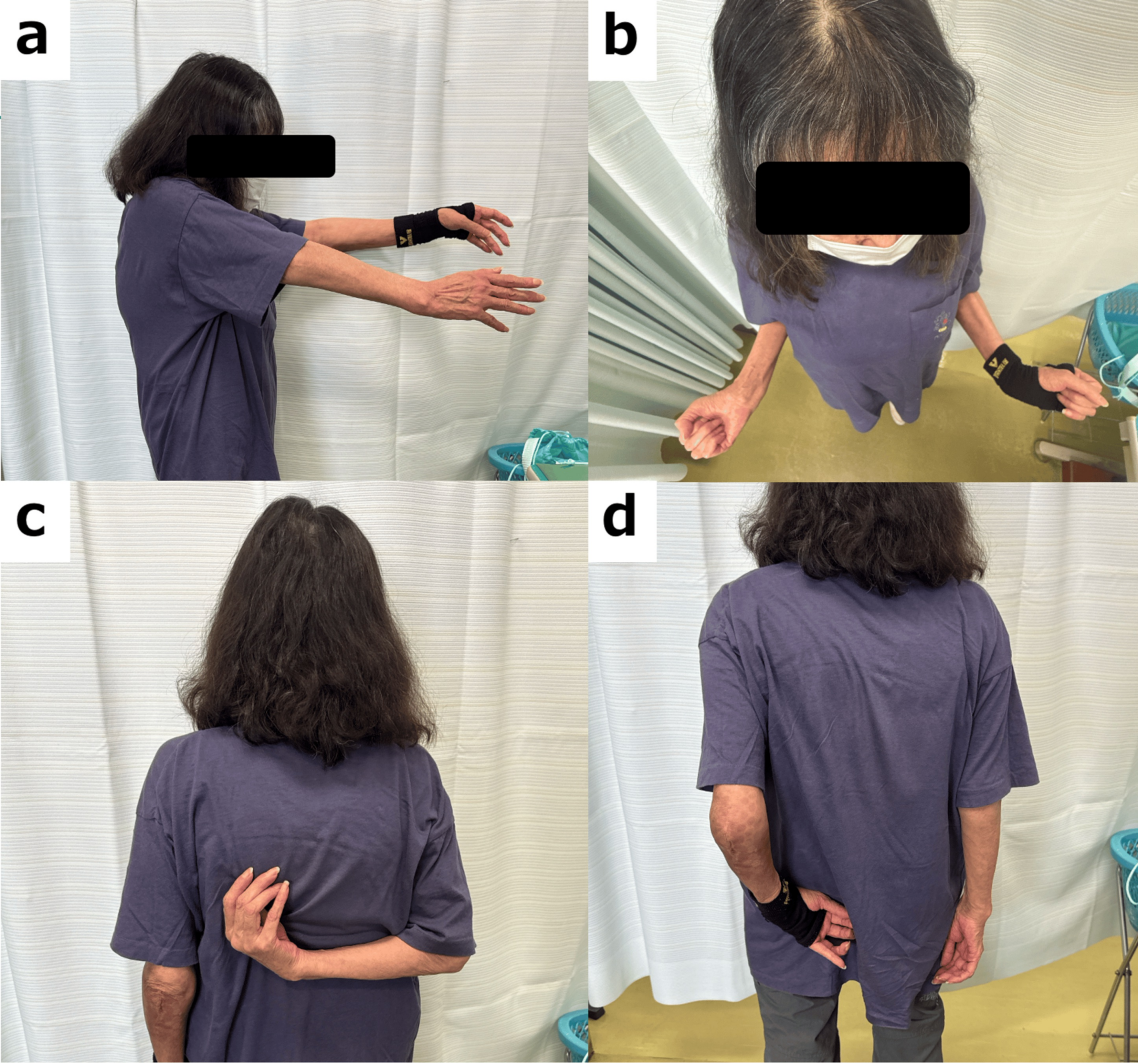
The final follow-up of shoulder function Postoperative function of both shoulders, including active elevation (a), external rotation (b), and internal rotation for the right (c) and left (d) shoulders.

At the final follow-up, while clinical outcomes had declined compared to those following the initial surgery, the patient had maintained a sufficient level of function for performing daily activities without significant limitations (Table 2, Figure 7).

**Table 2:** Comparison of functional outcomes between six months after primary surgery and final follow-up Range-of-motion values in degrees; internal rotation shown as spinal level. ASES: American Shoulder and Elbow Surgeons; L: left; R: right

	Six months after primary surgery	Final follow-up
Anterior elevation	110 (R); 60 (L)	70 (R); 90 (L)
Abduction	100 (R); 50 (L)	60 (R); 80 (L)
External rotation	30 (R); 30 (L)	20 (R); 20 (L)
Internal rotation	L5 (R); L5 (L)	T12 (R); sacrum (L)
Constant score	55 (R); 45 (L)	45 (R); 44 (L)
ASES score	75 (R); 75 (L)	63 (R); 75 (L)

## Discussion

The issue of how to manage complex PHFs in older patients, particularly three- or four-part fractures, remains controversial. Although ORIF has been the standard treatment, RSA is increasingly being selected because of its favorable functional outcomes in cases with osteoporosis and complex fractures [2]. While simultaneous bilateral RSA has previously been reported [7,8], we opted to perform staged bilateral RSA with a two-week interval in this case due to the patient’s poor general condition. Although the initial postoperative course was reasonable, the patient later developed bilateral periprosthetic fractures. With the increasing indications for RSA, periprosthetic humeral fractures have gained attention as clinically significant complications. These fractures, particularly those occurring around the tip of the cement, have been reported following approximately 3.5% of RSA procedures, and are associated with factors such as postoperative falls and underlying osteoporosis [9]. In our case, bilateral periprosthetic fractures occurred at the tip of the cement after staging bilateral RSA for PHFs. As highlighted by Brusalis et al. [9], careful postoperative monitoring and early management of osteoporosis are essential, particularly in high-risk patients. In the present case, the fractures were likely multifactorial in origin, with the fall itself as the primary trigger, along with additional contributing factors that may have included severe osteoporosis and a delay in anabolic agent initiation after surgery. The treatment strategy, whether conservative or surgical, should be guided by the fracture pattern and presence of implant loosening, as these factors critically influence clinical outcomes. The surgical strategy was determined based on the fracture classification, location, and the presence or absence of implant loosening. [9] When considering treatment options for periprosthetic fractures, revision surgery with a long-stem implant is recommended when fractures are unstable [10,11]. In this case, although the fracture pattern was simple, rigid fixation of the proximal fragment by ORIF would have been technically difficult due to the presence of a cemented stem. We considered the option of ORIF with bone grafting, but ultimately judged that revision with a long-stem prosthesis offers a more reliable solution in this case, given the poor bone quality and risk of re-fracture. In similar situations, the advantages and drawbacks of each approach should be weighed carefully. Since there was no stem loosening, the surgical technique was to split the humeral stem vertically (windowing), fix it using high-strength non-absorbable sutures with the Nice knot technique to prevent re-fracture of the humeral shaft during the revision, and then replace the long-stem implant [12,13]. Fortunately, the postoperative course was uneventful. On the left side, on the other hand, the displacement was minimal, but loosening was observed around the humeral stem. Since stem replacement is recommended in cases of stem loosening, we proposed stem revision arthroplasty as a treatment option, but since the patient judged the pain to be within the tolerable range, conservative treatment was selected.

At the final follow-up, elevation and abduction had improved, but both internal and external rotation on the left side had declined. This may have been partly due to the development of a left elbow contracture prior to follow-up. Contrastingly, despite the observed loosening around the stem, a favorable bone union was achieved with conservative treatment using teriparatide. Moreover, previous reports have also documented favorable outcomes with conservative management using teriparatide, supporting its efficacy in promoting fracture healing in similar cases [14].

In this case, shoulder function declined after revision RSA but was relatively preserved with conservative treatment, suggesting that conservative management may be preferable when feasible. Additionally, early pharmacologic intervention for bone fragility may help reduce the risk of such complications.

## Conclusions

Periprosthetic fractures can complicate RSA, particularly in patients with osteoporosis. Anticipating these risks is crucial in treatment planning. Early administration of bone anabolic agents may play a pivotal role in preventing fractures and improving healing in this population.
